# Differences in brain connectivity between older adults practicing Tai Chi and Water Aerobics: a case–control study

**DOI:** 10.3389/fnint.2024.1420339

**Published:** 2024-09-11

**Authors:** Ana Paula Port, Artur José Marques Paulo, Raymundo Machado de Azevedo Neto, Shirley Silva Lacerda, João Radvany, Danilo Forghieri Santaella, Elisa Harumi Kozasa

**Affiliations:** ^1^Hospital Israelita Albert Einstein, São Paulo, Brazil; ^2^Centro de Práticas Esportivas da Universidade de São Paulo – CEPEUSP, São Paulo, Brazil

**Keywords:** Tai Chi, longevity, self-regulation, fMRI, functional connectivity, mind–body, embodied cognition, Stroop

## Abstract

**Background:**

This study aimed to investigate the neural mechanisms that differentiate mind–body practices from aerobic physical activities and elucidate their effects on cognition and healthy aging. We examined functional brain connectivity in older adults (age > 60) without pre-existing uncontrolled chronic diseases, comparing Tai Chi with Water Aerobics practitioners.

**Methods:**

We conducted a cross-sectional, case–control fMRI study involving two strictly matched groups (*n* = 32) based on gender, age, education, and years of practice. Seed-to-voxel analysis was performed using the Salience, and Frontoparietal Networks as seed regions in Stroop Word-Color and N-Back tasks and Resting State.

**Results:**

During Resting State condition and using Salience network as a seed, Tai Chi group exhibited a stronger correlation between Anterior Cingulate Cortex and Insular Cortex areas (regions related to interoceptive awareness, cognitive control and motor organization of subjective aspects of experience). In N-Back task and using Salience network as seed, Tai Chi group showed increased correlation between Left Supramarginal Gyrus and various cerebellar regions (related to memory, attention, cognitive processing, sensorimotor control and cognitive flexibility). In Stroop task, using Salience network as seed, Tai Chi group showed enhanced correlation between Left Rostral Prefrontal Cortex and Right Occipital Pole, and Right Lateral Occipital Cortex (areas associated with sustained attention, prospective memory, mediate attention between external stimuli and internal intention). Additionally, in Stroop task, using Frontoparietal network as seed, Water Aerobics group exhibited a stronger correlation between Left Posterior Parietal Lobe (specialized in word meaning, representing motor actions, motor planning directed to objects, and general perception) and different cerebellar regions (linked to object mirroring).

**Conclusion:**

Our study provides evidence of differences in functional connectivity between older adults who have received training in a mind–body practice (Tai Chi) or in an aerobic physical activity (Water Aerobics) when performing attentional and working memory tasks, as well as during resting state.

## 1 Introduction

There is a growing interest in identifying effective strategies to promote healthy brain aging and preserve cognitive abilities (United Nations, 2019; World Health Organization, 2017). Among them, aerobic physical activities and mind–body practices play a fundamental role in preserving structural and functional health of the brain and they are widely acknowledged as protective factors for maintaining cognitive function and promoting healthy aging in older adults (Erickson et al., 2011; Song et al., 2017; Stillman et al., 2020; Erickson et al., 2022). They can exert beneficial effects on the brain via processes of neuroplasticity (e.g., changes in functional connectivity) which may converge into improvements in cognitive performance (Voss et al., 2013; Li et al., 2022). Tai Chi (a mind–body practice) and Water Aerobics (an aerobic physical activity) were the practices compared in the present study and they were described below in more detail.

Tai Chi is a mind–body practice that combines gentle flowing movements, with refined movement control; it includes controlled breathing, and focused attention, promoting a state of relaxed alertness and integrating the mind and body. As a result, Tai Chi has gained recognition for offering distinct advantages for both physical and mental health (Kong et al., 2019; Birdee et al., 2009) and enhancing brain function (Cui et al., 2021; Port et al., 2018; Yang et al., 2020). Studies have addressed its capacity to improve cognitive performance in older adults (Wayne et al., 2014). Moreover, mind–body practices like Tai Chi enhance interoception, which refers to the perception and awareness of bodily sensations (Farb et al., 2013; Farb et al., 2015). Interoceptive awareness and sensorimotor control are recognized as central to mind–body regulation (Khalsa et al., 2018; Quadt et al., 2018) and they play a significant role in Tai Chi training.

Water Aerobics also has benefits for health aging. It is an aerobic physical activity that stands out as an effective way to maintain a healthy body weight and prevent muscle and joint injuries (Tamin and Loekito, 2018). It is effective in treating patients with hemorrhagic stroke (Li and Chen, 2021), it has been associated with reduced anxiety and depression, improved functional independence, and decreased oxidative stress among older adults with depression (da Silva et al., 2019). Moreover, a systematic review has highlighted the benefits of Water Aerobics for patients with fibromyalgia (Bidonde et al., 2014). We would like to highlight that in contrast to Tai Chi interoceptive attentional focus, Water Aerobics involves repetitive movements and externalized attention focused on following the instructor’s motions. Furthermore, in Water Aerobics neuromuscular activity required from antigravity muscles is reduced (Pereira Neiva et al., 2018; Barbosa et al., 2009; Butts et al., 1991) while Tai Chi works with grounding or antigravity muscles. Besides, Tai Chi works with previous choreographed and memorized movements while Water Aerobics works with repetitive and copied movements. Therefore, Water Aerobics was chosen as an active control group for Tai Chi practice.

While one ages, changes occur in brain structure and function, including alterations in connectivity patterns within neural networks (Grady, 2012). These networks are shaped by the anatomical links among many brain regions, representing the exchange of information among them. Through functional brain connectivity, one can identifiy correlations between spatial regions of interest by employing linear temporal correlations based on parameters of neuronal activity (Xie et al., 2021; Smitha et al., 2017). Changes in functional connectivity have been utilized to identify age-related differences, with connectivity patterns within Salience Network (SN), Frontoparietal Network (FN), and Default Mode Network (DMN), serving as potential indicators of healthy or pathological brain aging (Buckner et al., 2005; Gardner et al., 2013; Seeley et al., 2009; Tomasi and Volkow, 2012; Zhou et al., 2012). Thus, engaging in exercise training and in practices that require centered attention and promote mind–body integration, such as Tai Chi may induce neuroplastic changes that could counteract age-related declines in memory, executive function, attention, abstract reasoning skills, and problem-solving abilities (Erickson et al., 2011; Tang et al., 2015; Tang et al., 2010).

Previous studies have shown that aerobic physical activities increase the size of hippocampus and improve memory (Erickson et al., 2011) and that mind–body practices, including meditation and movement meditation like Tai Chi, can influence brain networks associated with attention, emotional regulation, and cognitive control (Brewer et al., 2011). Mind–body practices have been linked to enhanced functional connectivity and structural changes in regions involved in self-referential processing and attentional control (Port et al., 2018; Tang et al., 2010; Kozasa et al., 2012). However, the neural mechanisms that distinguish mind–body practices from aerobic physical activities and elucidate their advantages in healthy aging remain unclear. Little is known about the differences that mind–body practices and physical practices may present on brain connectivity in adults over 60 years of age, without pre-existing diseases. This exploration might also aid in the development of targeted specific interventions to promote healthy aging and improve cognitive well-being in older adults. We observed differences in activation within regions linked to self-awareness, alongside enhanced efficacy in areas associated with attention and memory in the Tai Chi group compared to Water Aerobic group in a previous study, however a sample size of 16 participants did not allow robust conclusions (Port et al., 2018). Thus, in this study, which builds up on the findings of our previous study, we aimed on recruiting a higher number of participants.

Our hypothesis was that, due to the combination of conscious attention to movements and interoceptive training, Tai Chi practitioners would present heightened brain connectivity among areas linked to cognitive control, sensorimotor control, and interoception compared with Water Aerobics practitioners. This study aimed to explore differences in functional brain connectivity among older adults (age > 60) participating in interoceptive, attentional, and movement control training, specifically Tai Chi, or an aerobic physical activity, such as Water Aerobics. Participants performed N-Back and Stroop tasks during magnetic resonance imaging (MRI) scans.

## 2 Methods

### 2.1 Study design

This is a case control cross-sectional study conducted at the Brain Institute of Hospital Israelita Albert Einstein, São Paulo, Brazil. This work was approved by our institutional ethics committee (CAAE: 38602714.7.0000.0071).

Out of 57 volunteers contacted, 44 took part in the study (were evaluated), and among those, 32 met the pairing criteria. Both groups were paired in terms of sex, age, education, and the time of practice. After signing of the informed consent form, volunteers underwent neuropsychological assessment and then underwent functional magnetic resonance imaging.

### 2.2 Participants

To assure that characteristics of volunteers were compatible to inclusion and exclusion criteria, the researcher responsible for contacting the participants has also interviewed the Tai Chi and Water Aerobics instructors during data collection. We took special care to ensure that the chosen Tai Chi participants refrained from practicing any other mind–body techniques, such as yoga, various meditation styles, karate, or Qigong. Similarly, we ensured that the Water Aerobics volunteers were not involved in any of these other mind–body practices. Furthermore, we verified that the volunteers did not fall under the category of obesity, and they were carefully paired based on their individual gender, age, education and years of practice. While the stringent criteria and precise selection criteria led to the smaller sample size, they facilitated a controlled matching process, resulting in a distinct sample of older adults free from pre-existing conditions or with controlled chronic diseases trained in each activity.

#### 2.2.1 Inclusion criteria

Male or female aged>60; not obese (BMI < 30); who had practiced one of the two activities for at least 3 years, at least 1 h twice a week; non-practitioners of other mind–body activities; not colorblind; right-handed.

#### 2.2.2 Exclusion criteria

We excluded individuals who were interested in participating in the study if they had alterations or diseases involving the vestibular system, had uncontrolled chronic diseases such as diabetes or hypertension, had altered neurological conditions that resulted in spasticity or involuntary movement, had a neurological or psychiatric condition that hindered their ability to perform the tasks, were taking benzodiazepines or analgesic medications at doses that interfered with their attention span, had undergone orthopedic surgeries and/or received botulinum toxin injections in the last 6 months, met any criteria that contraindicated them from having a magnetic resonance imaging (such as having a cardiac pacemaker or cochlear implants), had dental artifacts, or had tremors or dystonia in the head and neck region.

The participants were interviewed by experienced nurses specialized in clinical studies to check the inclusion and exclusion criteria and after that they answered the questionnaires and scales.

### 2.3 Questionnaires and scales

The neuropsychological assessment was designed to probe whether group differences in memory skills, attention, executive functions, learning, recall skills, well-being and psychomotor efficiency. We conducted the assessment using the following tests in paper and pencil version:

**World Health Organization Well-Being Index (WHO-5)**: A brief questionnaire consisting of 5 simple, noninvasive, and positively phrased statements used to assess subjective well-being. Participants rated each statement on a 6-point Likert scale, ranging from 5 (all the time) to 0 (at no time), indicating how they felt during the last 2 weeks. The internal consistency is 0.78 in a Brazilian sample (Fleck et al., 2000).

**Pittsburgh Sleep Quality Index (PSQI)**: This scale evaluates sleep quality over the past 30 days through objective questions related to sleep schedule, nighttime awakenings, and daytime state. Each of the 19 self-reported items belongs to one of seven subcategories: subjective sleep quality, sleep latency, sleep duration, habitual sleep efficiency, sleep disturbances, use of sleeping medication, and daytime dysfunction. There are five additional questions, rated by the respondent’s roommate or bed partner, which are included for clinical purposes and are not scored. The Cronbach’s alpha in a Brazilian sample is 0.71 (Buysse et al., 1989).

**Self-Report Questionnaire-20 (SRQ-20)**: An inventory consisting of 20 questions used to detect psychiatric symptoms and assess mental health. The cutoff value was 7/8, with 86.33% sensitivity and 89.31% specificity. The score range is 0–20 (Scholte et al., 2011).

**Beck Depression Inventory (BDI)**: The BDI assesses depression symptoms using 21 items, with each item scored on a scale from 0 to 3. The total score range is 0–63, and a cutoff value of 20 points discriminates mild to moderate symptoms of depression, with a sensitivity of 0.77 and specificity of 0.95 (Beck et al., 1988b).

**Beck Anxiety Inventory (BAI)**: The BAI measures anxiety symptoms with 21 items, rated on a scale from 0 to 3, resulting in a total score range of 0–63. The internal consistency was 0.91, and the test–retest reliability was 0.99 for a sample of the Brazilian population (Beck et al., 1988a).

### 2.4 Neuropsychological tests

**Trail Making Test Part A and Part B**: Part A involves connecting 25 numbers, while Part B requires connecting numbers and letters in alternating ascending order using a pencil line. The examiner records the number of errors committed and the time taken to complete each task. They measure sustained and selective attention, respectively (Spreen and Strauss, 1998).

**Stroop Color and Word Test**: This paper-pencil task measures selective attention, cognitive flexibility, and the ability to suppress habitual responses in favor of unusual responses. The subject must quickly name the colors of four rectangles printed in green, blue, black, and red. The examiner records the number of errors and the time taken to complete the task (Spreen and Strauss, 1998).

**Rey’s Auditory Verbal Learning Test (RAVLT)**: The RAVLT is a paper-and-pencil measure of verbal memory for unstructured information (word lists). The examinee is presented with a list of 15 unrelated concrete nouns (List A) and is asked to recall as many words as possible. This process is repeated four more times, followed by the presentation and recall of List B. After approximately 25 min, the examinee is asked to recall List A again. The score is based on the number of words recalled at each stage. The estimated average application time for the first part (presentation of Lists A and B and immediate recall of List A) is 10 to 15 min, and the second part (delayed recall) takes approximately 10 min. The RAVLT provides 10 basic scores, including the number of words recalled in each attempt and the recognition of words from List A and List B (Salgado et al., 2011).

**Verbal Fluency F.A.S. (COWA)**: This test measures the ability to recall words within specific categories. The examinee is asked to generate as many words as possible starting with each of the letter’s F, A, and S within a specified time while following certain rules. The scores are obtained for each letter and a general score representing the total number of words evoked for the three letters (Spreen and Strauss, 1998).

**Mini Mental State Examination (MMSE)**: The MMSE assesses various cognitive functions through questions categorized into seven areas: time orientation, location orientation, three-word registration, attention and calculation, three-word recall, language, and visual construction capacity. The MMSE score ranges from 0 to 30. It is a screening tool for cognitive deficits. Its Cronbach alpha is 0.71 and kappa is 0.79 for a Brazilian sample (Bertolucci et al., 1994).

**Digit-Symbol (DS WAIS-III)**: This test evaluates processing speed, visual-motor response, and association between numbers and symbols within a 2-min duration. The score range is 0–133, and the test–retest reliability for the Brazilian sample was 0.84 (Wechsler, 2004).

### 2.5 Functional magnetic resonance imaging

All functional magnetic resonance imaging (fMRI) scans were performed using a 3.0 T “Discovery MR750w GE Healthcare®” equipment with a 32-channel head coil. The scans were conducted at the Department of Diagnostic and Preventive Medicine—Sector of Image of Hospital Israelita Albert Einstein. The NNL system (NNL, Norway) with a dedicated algorithm (Eprime) was used for stimulus presentation and acquisition of behavioral responses. This system utilizes independent binocular projection.

#### 2.5.1 Acquisition parameters

The fMRI evaluation was performed using paradigms prepared specifically for the evaluation of certain brain functions. The parameters of the acquisition sequences, sensitive to the BOLD effect (blood oxygen level dependent) were TR = 2000 ms, 40 slices with 3.3 mm thickness and 0.3 spacing between them. FOV = 240 mm, 64×64 matrix. The acquisition of the N-Back paradigm task had a total of 104 volumes (duration 3 min 28 s), and that of the Stroop task, 154 volumes (duration 5 min 8 s), and Resting-State had a total of 200 volumes (6 min 40 s).

T1-weighted Volumetric SPGR with axial acquisition, TR = 5 mss, TE = 1.7 ms, Matrix: 256 × 256, FOV 21.6 cm, Flip angle: 7, Thickness: 1.0 mm with 176 slices covering the entire cephalic segment, (duration 6 min 20 s). Axial FLAIR: TR = 1,000 ms, TE = 140 ms, IR = 1000s, FOV = 24 cm, Thickness = 5 mm, Spacing = 2.5 mm, 20 levels, for potential identification of other co-morbidities, t = 4:40s.

#### 2.5.2 Description of tasks for functional assessment

Participants familiarized themselves with the tasks prior to the fMRI sessions and were scanned while performing adapted versions of the Stroop Word-Color Task (SWCT) and N-Back task. The SWCT was presented before the N-Back task.

##### 2.5.2.1 Attention paradigm (Stroop word—color task)

The SWCT is a classic task used to study attention and inhibition and has been used in fMRI as a paradigm for understanding cognitive control mechanisms (Peterson et al., 1999). During this task, participants were instructed to communicate the color (blue, red, or green) of isolated words by pressing one of the three buttons, corresponding to these colors. Words were presented in three conditions: congruent (for example, the word “BLUE” painted in blue), neutral (color and words are not related), and incongruent (for example, the word “BLUE” painted in green). Each condition was presented in five blocks of trials, with 10 trials within each block. Each word/stimulus was presented for 1 s, with 1 s of inter-stimulus interval. Conditions were presented sequentially, without rest in between them, and always in the following sequence: congruent, neutral and incongruent. There were 8 s of waiting period before the first block of trials for a total of 154 TRs (5 min and 8 s).

##### 2.5.2.2 N-Back task memory paradigm

The N-Back task has been frequently used in the investigation of the neural correlates of the working memory process (Owen et al., 2005). In the “2-back” (remember) condition, the target letter was a repeated letter, separated by another letter. The “0-back” (find) condition is a baseline task to control attention and motor movement, where participants were instructed to respond whenever the letter ‘X’ was displayed. Each condition was presented in five blocks of trials, each with 10 trials, alternating between “0-back” and “2-back” conditions. No rest periods were introduced between these blocks. Letters were presented for 1 s, with a 1 s inter-stimulus interval. There were 8 s of waiting period before the first block of trials for a total of 104 TRs (3 min 28 s).

#### 2.5.3 Data analysis

##### 2.5.3.1 Activation data analysis

Functional activation analysis was performed using the FSL program (Oxford, University 2010. FSL, Oxford England) which provides several routines for processing fMRI images.

The processing of the FMR images was performed with the FEAT tool (FMRI Expert Analysis Tool), version 6.0 integrated with the FSL software (FMRIB’s Software Library). The following image pre-processing sequence was performed: the first four initial volumes were removed because they represented acquisition calibration parameters, two step high-resolution registration to the MNI152 2 mm standard space image is carried out using FLIRT (Jenkinson et al., 2002; Jenkinson and Smith, 2001) affine registration with 12 degrees of freedom, motion correction using MCFLIRT (Jenkinson et al., 2002) removal of non-brain tissue with the BET tool (Smith, 2002) slice-timing correction using Fourier-space time-series phase-shifting, spatial smoothing with a Gaussian kernel of FWHM 5 mm, intensity normalization of the general mean in the 4D dataset by a single multiplicative factor, high-pass temporal filtering (Gaussian-weighted least-squares straight line fitting, with sigma = 100 s). Time-series statistical analysis is carried out using FILM with local autocorrelation correction (Smith, 2002). The recording of the functional image on the high-resolution T1-weighted anatomical image and the subsequent normalization to the standard brain Montreal Neurological Institute (MNI) 152, with 2 mm resolution were performed with the FLIRT tool (Jenkinson et al., 2002; Jenkinson and Smith, 2001).

##### 2.5.3.2 Functional connectivity data analysis

Data pre-process and analysis was conducted using CONN Toolbox 20.b version standard pipeline and parameters. We conducted functional connectivity analysis on the Resting-state, Stroop and N-Back fMRI data without segmenting for each stimulus condition. Consequently, the Stroop task analysis incorporated data from congruent, incongruent, and neutral conditions, while the N-Back analysis included the 0-back and 2-back conditions. We made this choice because there were insufficient data points in the fMRI data to perform connectivity analyses for each stimulus separately (Parkes et al., 2018). Operational procedure consisting in realignment, unwarping deformation, slice time correction, segmentation, normalization, *outliers’* detection and smoothing. Confounding variables were based on head movement (discarding volumes with displacement >2 mm and global signal z-value >9; no subjects were excluded), realignment parameters, white matter, and cerebrospinal fluid signals. Bandpass filtering (0.008 to 0.09 Hz) was applied to filter out physiological noise, and confounding variables were removed using a simultaneous bandpass approach. For this functional connectivity study, we conducted a seed-to-voxel analysis, using Salience and Frontoparietal networks as seeds. We did a functional connectivity analysis between the seed regions and the whole brain voxels. We set a *p*-value of 0.05 (FDR-corrected) for significant differences between groups.

##### 2.5.3.3 Regions of interest

We used Salience network including Anterior Cingulated Cortex (x = 0, y = 22, z = 35), left Anterior Insular Cortex (x = −44, y = 13, z = 1), right Anterior Insular Cortex (x = 47, y = 14, z = 0), left rostral Prefrontal Cortex (x = −32, y = 45, z = 27), right rostral Prefrontal Cortex (x = 32, y = 46, z = 27), left Supramarginal Gyrus (x = −60, y = −39, z = 31) and right Supramarginal Gyrus (x = 62, y = −35, z = 32).

The Frontoparietal Network included left Lateral Prefrontal Cortex (x = −43, y = 33, z = 28), right Lateral Prefrontal Cortex (x = 41, y = 38, z = 30), left Posterior Parietal Lobe (x = −46, y = −58, z = 49) and right Posterior Parietal Lobe (x = 52, y = −52, z = 4; Whitfield-Gabrieli and Nieto-Castanon, 2012). All networks and ROIs were defined based on networks provided by the CONN Software.

### 2.6 Statistical analysis

#### 2.6.1 Demographic and neuropsychological statistical analysis

We used the Mann–Whitney’s test to analyze differences between groups in age, weight, height, body mass index and practice time. For the schooling category (elementary school, high school, under degree and graduate) we used a Fisher’s exact test to compare whether both groups were paired in terms of schooling. To test differences between groups in neuropsychological assessment, we used Mann–Whitney’s test. We set a *p*-value of 0.05 for statistical difference between groups. We conducted the statistical analysis using JASP Team (2024).

#### 2.6.2 Behavioral statistical analysis

We conducted an analysis of variance (ANOVA) to test whether the groups differed in terms of response time (in milliseconds) and accuracy (ratio of correct answers to total attempts) in the Stroop and N-Back tasks. Only attempts with correct answers were considered for the response time calculation. We set a p-value of 0.05 for statistical differences in the interaction between groups and stimuli, corrected by Bonferroni. *Post-hoc* analyzes were performed to describe variables between groups. Behavioral statistical analyses were conducted using JASP (Version 0.19.0).

#### 2.6.3 Activation statistical analysis

**Figure 1:**
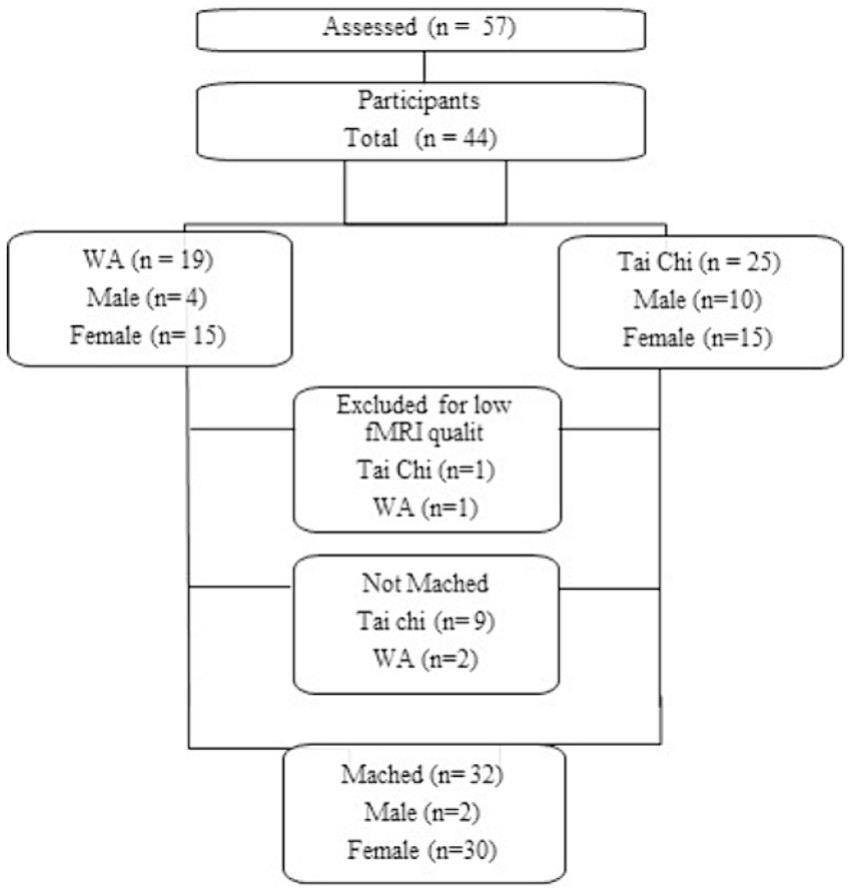
Study flowchart.

Statistical analysis was based on the massive univariate approach, using a General Linear Model (GLM), modeling the response in each voxel to the experimental conditions of the task. The activity of each of the two conditions of interest (Stroop: incongruent and neutral; N-Back: remember) were modeled as blocks of activity. The regressor for each of the conditions was modeled with a custom function, with duration established through the average of the trials of each task, inserted in a file containing three-column regressors. Next, each regressor was convoluted with a gamma function (time to peak = 5 s, standard error = 2.8 s). In addition to the repressors of interest, head movement parameters were also included as repressors of non-interest. In each participant, the contrast between the remember and find conditions was performed (remember > find for the N-Back task). For the Stroop task, the baseline was established by the congruent condition, resulting in contrasts incongruent > neutral and neutral> incongruent. It was evaluated using a t test for one sample, and the t values converted to Z. The group analysis was performed with a mixed effects model, using FLAME (FMRIB’s Local Analysis of Mixed Effects; Woolrich et al., 2001). Differences between groups were assessed by a two-tailed t test for independent samples for each of the contrasts. To control for false positive results, the Z statistic maps were initially thresholded using voxel clusters determined by Z > 3.1, and the cluster corrected significance threshold of *p* = 0.05 (Figure 1).

## 3 Results

### 3.1 Demographic and neuropsychological results

Table 1 shows demographic comparison between groups. There were no significant differences in age, year of practice and height. We found significant difference in Body Mass Index and Weight, however none of the participants were obese (BMI < 30). There were no differences in level of education (*p* = 0.139) and most of participants had a college degree. We found no significant differences between groups in the neuropsychological tests (BAI, BDI, COWA, MMSE, RAVLT, PSQI, Who-5, Stroop, Trail Making, Digit-Symbol and SRQ-20). Descriptive and inferential statistics regarding each task and group is available as Supplementary Table 1.

**Table 1:** Demographic results comparison between Tai Chi and Water Aerobics practitioners.

	Group	N	Mean	SD	*p*-value	Cohen’s D
Years of Practice	1	16	13.750	10.976	0.108	0.584
	2	16	8.563	6.099		
Age	1	16	68.063	6.658	0.583	0.066
	2	16	68.500	6.491		
Hight	1	16	1.576	0.077	0.151	0.449
	2	16	1.611	0.079		
Weight	1	16	57.938	7.646	< 0.001	1.448
	2	16	70.250	9.277		
BMI	1	16	23.386	3.075	0.002	1.256
	2	16	27.046	2.745		

BMI, Body Mass Index; 1, Tai Chi group; 2, Water Aerobics; SD, Standard Deviation.

### 3.2 Behavioral tests performed in fMRI

**N-Back Task**: We found no significant interaction effects between groups and stimuli in the ANOVA analysis for either accuracy (*p* = 0.338) or response time (*p* = 0.951). Detailed descriptive statistics (mean and standard deviation) for each group and stimulus, along with the corresponding inferential statistics (*p*-values and effect sizes), are provided in Supplementary Table 2.

**Stroop Task:** The ANOVA analysis of the Stroop Word Color Task revealed no significant interaction effects between groups and stimuli for either accuracy (*p* = 0.582) or response time (*p* = 0.29). Comprehensive descriptive statistics (mean and standard deviation) for each group and stimulus, as well as the corresponding inferential statistics (*p*-values and effect sizes), can be found in Supplementary Table 3.

### 3.3 fMRI activation analysis in N-Back and Stroop task

There were no differences between groups in the activation results. Notably, during Stroop task, we found consistent activation in occipital and frontal cortex areas. When comparing incongruent and neutral conditions, we identified a greater number of activation clusters, indicating the presence of the Stroop effect. (Supplementary Table 3 for N-Back; Table 2 for Stroop).

**Table 2:** fMRI activation analysis for Stroop task, no comparison between groups.

Contrast	Cluster	Index	Voxels peak	x	y	z	Area
Tai Chi (neutro>congruent)	4	191	4.07	−50	4	30	64% Precentral Gurys
	3	161	3.82	−44	−66	−10	41% inf. Lateral Occiptal Cortex
	2	159	4.07	8	−64	−12	45% R Cerebellum VI
	1	130	4.29	−64	−4	0	24% Superior Temporal Gyrus
WA (neutro>congruent)	No siginficant						
Tai Chi (incongruent>congruent)	4	12,891	5.51	−38	−38	40	27% Supramarginal Gyrus
	3	436	4.32	18	−72	58	58% Lateral Occiptal Cortex
	2	372	5.04	38	2	54	47% Middle Frontal Gyrus
	1	297	4.35	46	10	32	31% Precentral Gyrus
WA (incongruent>congruent)	2	392	4.29	−26	−96	16	61% Occiptal Pole
	1	201	4.2	−30	−58	46	33% Superior Parietal Lobule
Tai Chi [incongruent>congruent] > [neutro> congruent]	6	5,346	4.9	0	−64	52	60% Precuneous Cortex
	5	1,269	4.73	42	20	24	23% Middle Frontal Gyrus
	4	369	4.15	−42	12	32	44% Middle Frontal Gyrus
	3	231	4.28	56	−46	12	36% Middle Temporal Gyrus
	2	173	4.25	0	20	50	26% Superior Frontal Gyrus
	1	162	4.13	−36	2	54	44% Middle Frontal Gyrus
WA [incongruent>congruent] > [neutro>congruent]	2	260	4.53	14	−90	0	18% Occiptal Pole
	1	145	3.83	−36	−56	50	30% Superior Parietal Lobule

The table shows the clusters (voxels) that had activation by group, during task conditions for Stroop. Coordinates (x, y, z) are in MNI space. WA, Water Aerobics.

### 3.4 Functional connectivity comparison between groups: results for N-Back and Stroop tasks

#### 3.4.1 Resting state

**Figure 2:**
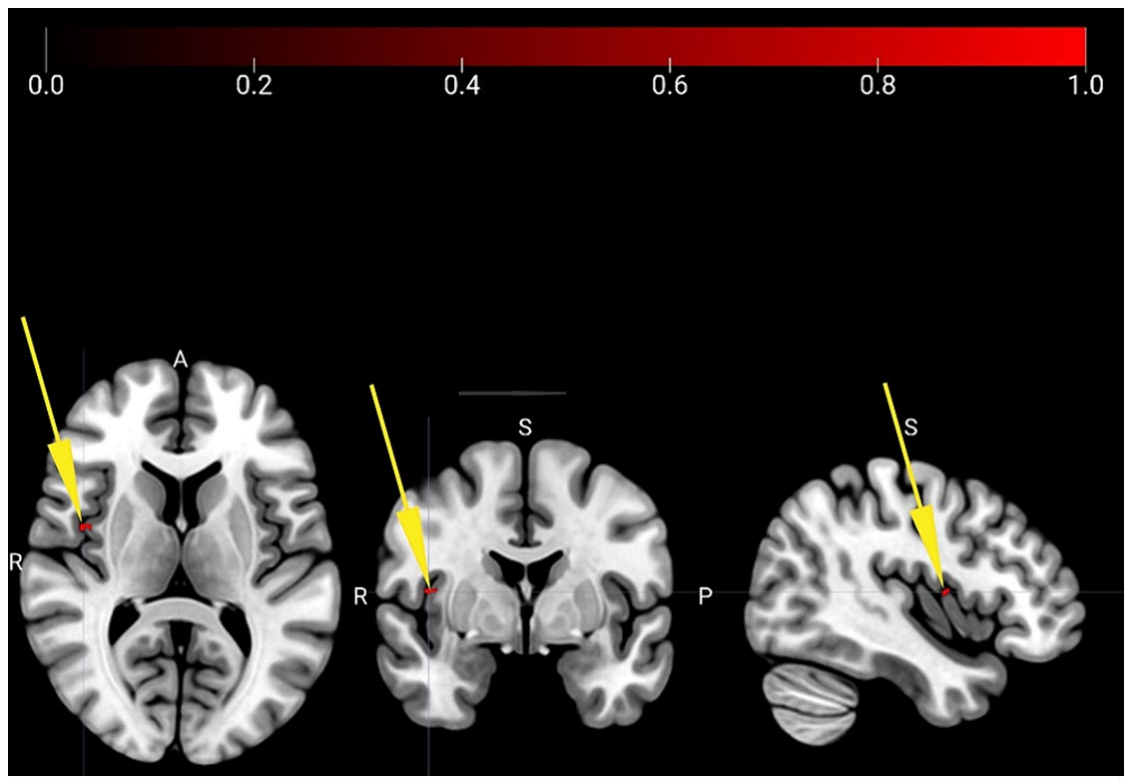
Resting State connectivity analysis comparison between groups. Tai Chi group exhibited an increased correlation between the seed of anterior cingulate cortex with the clusters central opercular cortex right and insular cortex right. Peak-cluster shown in red. Yellow arrows indicate localization of peak-cluster in MRI space (x = 42, y = 02, z = 18).

**Tai Chi > Water Aerobics**: During Resting State, using the salience network as seed, Tai Chi group exhibited a higher correlation of Anterior Cingulate Cortex (ACC) with two clusters, one covering the Right Central Opercular Cortex: 4 voxels (57%) and the other in the right Insular Cortex: 3 voxels (43%; Figure 2; Table 3).

**Table 3:** Connectivity comparison between Tai Chi and Water Aerobics groups.

Task	Seed	Correlation	Contrast	Cluster (x, y, z)	Size *p-*FDR
Resting State	Salience Anterior Cingulate Cortex	Central Opercular Cortex right: 4 voxels (57%) covering 0% of Central Opercular Cortex right Insular Cortex right: 3 voxels (43%), covering 0% of Insular Cortex right.	TC > WA	(+42, +02, +18)	0.040456
N-Back	Salience Left Supramarginal Gyrus	Cerebellum Crus 2 left: 49 voxels (42%) covering 3% of Cerebellum 2 left Cerebellum 7b left: 19 voxels (Farb et al., 2015) covering1% of Cerebellum 7b left Cerebellum Crus2 right: 12 voxels (10%) covering 15 of Cerebellum Crus2 right	TC > WA	(−4, −76, −38)	0.021126
Stroop	Salience Left Rostral Prefrontal Cortex	Occipital Pole right: 312 voxels (44%) covering 12% of Occipital Pole right Inferior Lateral Occipital Cortex right: 205 voxels (29%) covering 10% of Inferior Lateral Occipital Cortex right Superior Lateral Occipital Cortex right: 114 voxels (16%) covering 2% of Superior Lateral Occipital Cortex right	TC > WA	(+16, −94, −04)	0.0000001
	Frontoparietal Left Posterior Parietal Lobe	Cerebellum Crus 1 Left: 226 voxels (73%) covering 10% of Cerebellum Crus 1 Left Cerebellum Crus 2 left: 83 voxels (27%) covering 4% of Cerebellum Crus 2 left Cerebellum 6 left:2 voxels (1%) covering 0% of Cerebellum 6 left	WA > TC	(−30, −70, −32)	0.000311

The table shows the clusters (voxels) that had correlation differences, in the comparison between the groups and the nodes within the networks chosen a priori. TC refers to Tai Chi group and WA refers to Water Aerobics group. The networks chosen were the Salience and Frontoparietal networks. *p*-FDR refers to value with False Discovery Rate correction. Coordinates (x, y, z) are in MNI space.

#### 3.4.2 N-Back task

**Figure 3:**
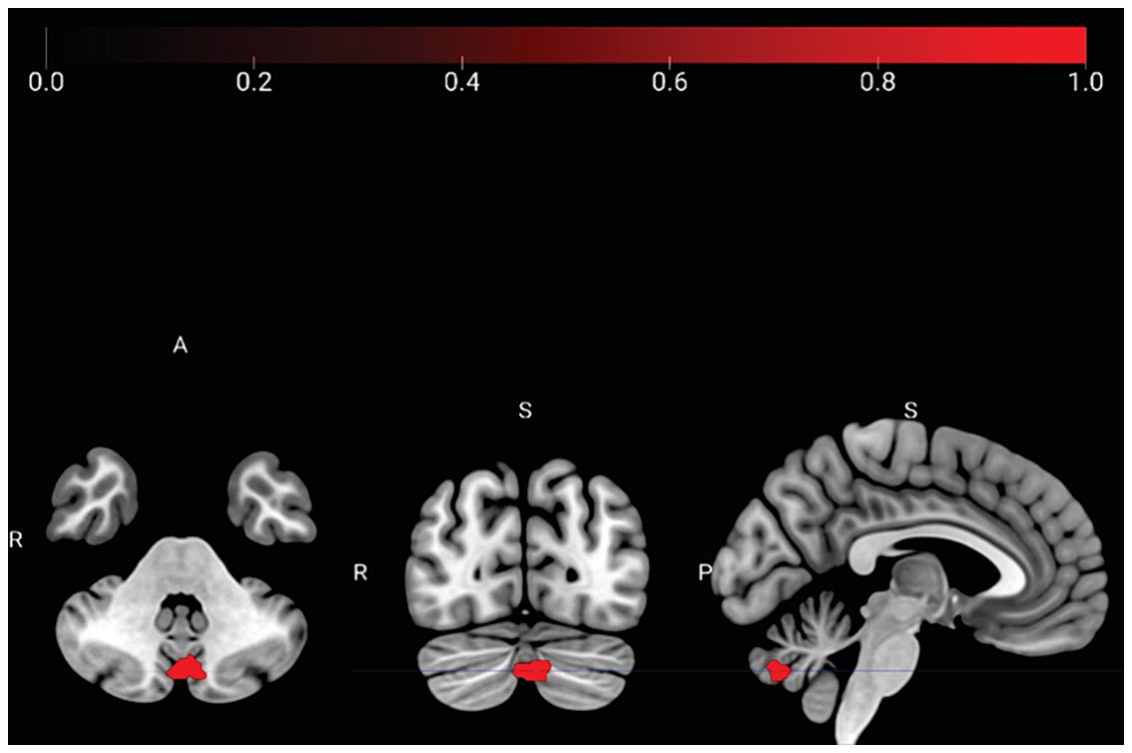
N-Back connectivity analysis comparison between groups. The Tai Chi group exhibited an increased correlation between the seed of left supramarginal gyrus and the clusters of cerebellum crus 2 left, cerebellum 7b left and cerebellum crus 2 right. Peak-cluster shown in red with coordinates in MNI space (x = −4, y = −76, z = −38).

**Tai Chi > Water Aerobics**: During the N-Back using salience network as seed, Tai Chi group showed a higher correlation of left Supramarginal Gyrus with three clusters, one covering the Cerebellum Crus 2 left: 49 voxels (42%), other covering Cerebellum 7b left: 19 voxels (16%) and Cerebellum Crus 2 right: 12 voxels (10%; Figure 3; Table 3).

#### 3.4.3 Stroop task

**Figure 4:**
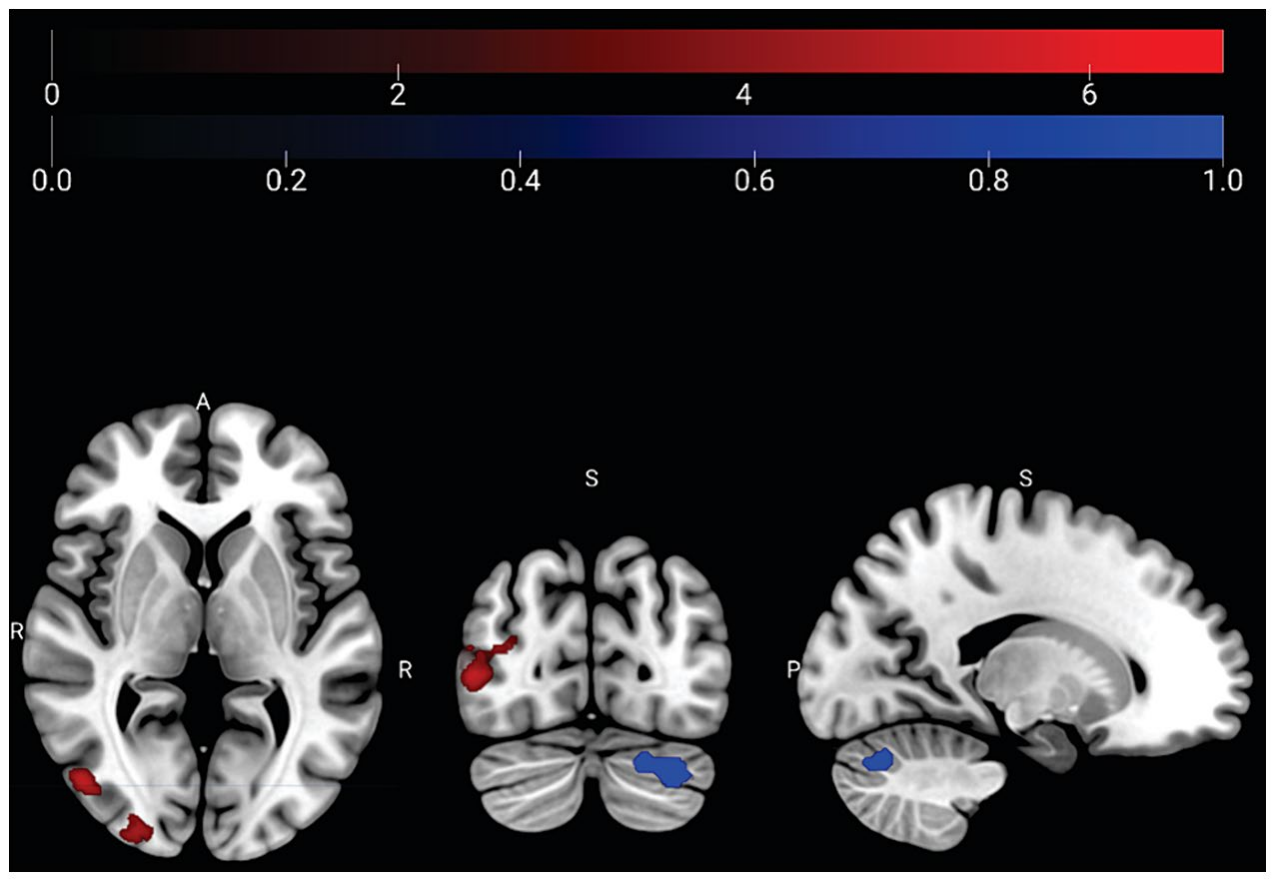
Stroop connectivity analysis comparison between groups. Using salience network, the Tai Chi group exhibited an increased correlation between the seed of the rostral left prefrontal cortex and the right occipital pole, the anterior division of the right lateral occipital cortex, and the superior division of the right lateral occipital cortex. Peak cluster shown in red at x = 16, y = −94, z = −04 in MNI space. Using frontoparietal network, Water Aerobics group exhibited increased correlation between the seed of the left posterior parietal lobe and the cerebellum crus 1 left, cerebellum crus 2 left, and cerebellum 6 left. Peak clusters shown in blue at x = −30, y = −70, z = −32.

**Tai Chi > Water Aerobics**: During the Stroop task using Salience Network as seed, Tai Chi group demonstrated a higher correlation of the Left Rostral Prefrontal Cortex with the right occipital pole: 312 voxels (44%), and inferior division of the right lateral occipital cortex: 205 voxels (29%), and the superior division of the right lateral occipital cortex: 114 voxels (16%). **Water Aerobics > Tai Chi**: Additionally, during the Stroop task using Frontoparietal network as seed, Water Aerobics group exhibited a higher correlation of the left posterior parietal lobe with 3 cerebellar clusters, Cerebellum Crus 1 left: 226 voxels (73%), Cerebellum Crus 2 left: 83 voxels (27%), and Cerebellum 6 left: 2 voxels (1%; Figure 4; Table 3).

## 4 Discussion

Our study investigated differences in functional activity and connectivity in adults over the age of 60, without reporting uncontrolled pre-existing chronic diseases, involved in mind–body training (Tai Chi), compared with adults of the same age involved in an aerobic physical activity (Water Aerobics), with a strict matching criterion for gender, age, education, and time of practice. There were no differences in neuropsychological tests and these results confirm that there were no statistically significant differences in measures of cognitive performance between groups. This concern in pairing allowed us to have as similar active groups as possible, valuing the observed differences. The recruitment process was therefore, challenging.

At first participants underwent neuropsychological tests, then were assessed during a working memory task (N-Back task) and an attention and inhibitory control task (Stroop task). We performed a seed-to-voxel analysis using Salience and Frontoparietal networks as seeds.

Contrary to our expectations, our findings revealed no behavioral variances between groups in either the Stroop or N-Back tasks. The groups’ shared characteristics of good health, comparable education (years of schooling), and the same amount of physical exercise (engaging in physical activity for over 3 years, at least twice weekly). This outcome indicates the study’s efficacy in reducing inter-group variations.

### 4.1 Brain activation

Our investigation into brain activation revealed no discernible differences between groups during cognitive testing, which contradicts our initial hypothesis. These results are particularly notable given a prior study that identified differences in brain activation between Tai Chi and Water Acrobatics groups within a subset sample (Port et al., 2018). However, our current findings suggest a uniformity in brain activation across groups during task performance, hinting at potential similarities in neural processing despite differences in training interventions. Future studies with a larger sample size are necessary to buttress (or refute) our observations.

Despite the absence of group differences in brain activation, our analysis shows consistent activation patterns in the Stroop task in both groups. Notably, we observed activation in both occipital and frontal cortex areas, known for their involvement in visual processing and executive control functions, respectively (Huang et al., 2020). This observation aligns with existing literature on the neural correlates of the Stroop task, supporting the reliability of our neuroimaging methodology. Despite the absence of group differences in brain activation, our analysis shows consistent activation patterns in the Stroop task in both groups. Notably, we observed activation in both occipital and frontal cortex areas, known for their involvement in visual processing and executive control functions, respectively (Huang et al., 2020). This consistency aligns with existing literature on the neural correlates of the Stroop task, supporting the reliability of our neuroimaging methodology.

### 4.2 Connectivity

#### 4.2.1 Resting state

Supporting our initial hypothesis, our findings revealed that Tai Chi group presented increased connectivity between Anterior Cingulate Cortex (ACC), Insular Cortex (IC), and Central Opercular Cortex (insular area) during resting state, compared to Water Aerobics group. The Central Opercular Cortex, along with Frontoparietal areas, is involved in cognitive control (Marek and Dosenbach, 2018). The Anterior Cingulate Cortex and Insular Cortex are crucial components of Salience Network, consistently co-activated in response to salient stimuli, whether external or internal, irrespective of their emotional valence (Craig, 2009a). These regions play a significant role in interoceptive processes and, consequently, in physiological regulation (Chong et al., 2017). For example, the ACC is involved in both bodily arousal and interoceptive accuracy and sensitivity. Accuracy refers to the objective precision of bodily events such as heartbeats and temperature, while interoceptive sensitivity is the subjective interpretation of internal states of the body such as restlessness, confusion, and calmness (Pollatos et al., 2007). In turn, Insular Cortex, especially the Anterior Insular Cortex (AIC), is crucial for integrating internal and external signals and plays a role ranging from affective and sensory processing to higher cognitive processes (Menon et al., 2022). According to Craig (2003) the AIC performs a meta-representation of primary afferent interoception in humans, forming the basis for the subjective image of the “self,” for feelings, cognitive awareness, and emotions (Marek and Dosenbach, 2018).

Connections between the ACC and IC appear to play a central role in sensorimotor, affective, and cognitive interplay and are crucial for the link between body and subjectivity, interoception, and self-regulation. Disruptions in connectivity of these areas are associated with emotional fragility and affective dysregulation implicating psychiatric disorders such as anxiety, panic and narcissistic personality disorder (Schimmelpfennig et al., 2023; Stein et al., 2007; Pannekoek et al., 2013). Additionally, as central areas in salience network, the ACC and IC act as a filter between internal and external stimuli, which is related to maintaining goal-directed behavior (Menon, 2011). Changes in connectivity between these areas can compromise the switching of activation between default mode network (DMN) and executive control network (FN), leading to alterations in cognitive function and age-related dysfunctions (Menon, 2011).

Our results indicated increased connectivity between the ACC and IC in Tai Chi group, corroborating previous studies showing that Tai Chi is associated not only with plasticity in functional brain networks during resting state but also with changes in insular connectivity (Cui et al., 2021; Xu et al., 2020; Yue et al., 2020).

Consistent with other studies, we found that combined body and cognition training, as observed in Tai Chi, is associated with increased brain connectivity between areas that are particularly relevant to interoceptive accuracy during rest (Chong et al., 2017). This training is also related to areas involved in regulation of body physiology (Duquette, 2017) self-regulation skills, cognitive control, and motor organization of subjective aspects of experience through IC and ACC (Craig, 2011; Craig, 2009b; Vago and Silbersweig, 2012).

#### 4.2.2 N-Back

Our findings revealed a stronger correlation between the left Superior Marginal Gyrus (LSMG) and cerebellum (Crus I and II) in Tai Chi group compared to Water Aerobics group during N-Back task, using Salience Network as seed region. The LSMG is known to play a causal role in cognitive processing and is a key area in short-term memory network, crucial for retention and sequencing of abstract representation information (Guidali et al., 2019). Moreover, the LSMG is associated with temporal sequencing of movement, adaptation to environmental changes, sustaining attention, sequential memory retrieval, and embodiment (Cabeza et al., 2008; Hartwigsen et al., 2012).

Tai Chi involves slow and choreographed movements performed in specific sequences and rhythms, synchronized with breathing, and requires attentional regulation and movement awareness, and this cognitive aspect of Tai Chi is likely responsible for the increased connectivity observed in the LSMG of the Tai Chi group (Jiang et al., 2021).

Cerebellum is known to contribute to affectivity by regulating emotions, as well as the rhythm and precision of movement (Guell et al., 2018). It plays a crucial role in psychophysiological regulation, as motor effort and movement qualities are essential factors (Balasubramaniam et al., 2021). Increased connectivity between the LSMG and cerebellum suggests that conscious movement, a characteristic of Tai Chi and other contemplative practices involving movement, may be associated with this type of regulation.

Recent research on the cerebellum highlights its integration with interoceptive training and sensorimotor control. Crus I of the cerebellum is associated with mirroring, while Crus II is specialized in mentalizing, social cognition, and emotional self-perception (Van Overwalle et al., 2014; Van Overwalle et al., 2020). Additionally, cerebellum is involved in complex functions such as strategy formation, cognitive flexibility, and working memory (Schmahmann, 2019). It modulates cognition like it modulates the motor system and is implicated in interoceptive sensitivity (Smith et al., 2022).

#### 4.2.3 Stroop

In the Stroop task group comparison, salience network seed exhibited a more robust correlation with the Left Rostral Prefrontal Cortex (R-PFC), Right Occipital Pole, Right Lateral Occipital Cortex (Anterior Division), and Superior Division of the Right Lateral Occipital Cortex in Tai Chi group in contrast to Water Aerobics group.

Previous studies suggest that the Rostral Prefrontal Cortex (R-PFC) plays a role in sustaining attention and prospective memory, and it is known to mediate attention between external stimuli and internally maintained intentions (Benoit et al., 2012; Burgess et al., 2000). Occipital pole is responsible for integration and perception of visual information and integrates the visual association cortex, which interprets visual images (Rehman, 2022). In a review, posterior occipital and temporal areas were also associated with selective attention (Cabeza and Nyberg, 2000). These functions are important for inhibitory control and stimulus recognition, which are relevant to Stroop task. Other studies have linked visual areas of the brain to interoceptive sensitivity (Smith et al., 2022; Kuehn et al., 2016). In addition, interoception has been associated with prefrontal cortex activation and attention regulation (Vago and Silbersweig, 2012).

The increased correlation between prefrontal and visual areas involved in the integration of internal and external information, inhibitory control, and stimulus recognition in Tai Chi group, during Stroop task, is likely to the fact that Tai Chi practice encompasses elements of meditation and martial art. During Tai Chi, internal attention is sustained while simultaneously maintaining external attention, along with the execution of slow movements based on attack and defense actions. Thus, Tai Chi integrates attention to both internal states and external factors, involving salient stimulus discrimination (or imminent danger) and internal impulse control directed toward effective action (attack or defense). These findings support Tai Chi as a cognitive training for selective attention, sustained attention, and inhibitory control. Additionally, Tai Chi encompasses movements for combat training, enhancing bodily awareness, potentially optimizing sensorimotor regulation of affective states (Critchley, 2005). Interoception also appears to be involved in the development of these regulatory functions (Critchley et al., 2004).

When using Frontoparietal network as seed for Stroop task, Water Aerobics group showed a stronger correlation between the Left Posterior Parietal Lobe (LPPL) and Crus I and Crus II of cerebellum compared to Tai Chi group. The LPPL is the region consistently activated in functional neuroimaging studies related to conceptual processing (Binder et al., 2009). In conceptualization tasks, such as word comprehension, the LPPL acts convergently, integrating information from various perceptual modalities (Damasio, 1989). This suggests that Water Aerobics group utilized a less specialized and more generalized perception in the attention task, unlike Tai Chi group, which exhibited correlations with interoceptive awareness areas (sensitivity, discrimination, and accuracy).

Water Aerobics group showed a stronger correlation between the LPPL and Crus I of cerebellum. The LPPL is specialized in representing motor actions and motor planning directed at objects (Kiefer and Pulvermüller, 2012). And Crus I is an area of cerebellum linked to object mirroring (Schmahmann, 2019). This object mirroring suggests an externally focused strategy, a motor representation strategy based on shape recognition related to word meaning (Kuhnke et al., 2020). This could be attributed to Water Aerobics emphasis on imitating the instructor’s movements. Our results indicate that, to perform an attention and conflict resolution task, Water Aerobics group exhibited increased connectivity between areas related to mirroring and exteroception, while Tai Chi group showed increased connectivity between areas involved in sustained attention, prospective memory, and interoceptive sensitivity.

### 4.3 Limitations

Although we revealed differences in brain connectivity between Tai Chi and Water Aerobics during Resting State, N-Back and Stroop tasks, some limitations must be addressed. We did not have a control group without practicing physical activities, which probably had health issues. We had a small sample size due to our strict group matching criteria which allowed us to compare participants with similar characteristics beyond their practices. This rigid pairing values the specificity of the differences found. Due to the cross-sectional design of our study, we cannot establish causal relationships between interventions and outcomes, precluding claims on which type of intervention may exert more pronounced effects on brain function.

## 5 Conclusion

Our study provides evidence for differences in functional connectivity patterns between older adults who have engaged in a mind–body practice (Tai Chi) or in an aerobic physical activity (Water Aerobics) when performing attentional and working memory tasks, as well as during resting state.

## Data Availability

The raw data supporting the conclusions of this article will be made available by the authors, without undue reservation.
