# Supplementary material for: Differences in brain connectivity between older adults practicing Tai Chi and Water Aerobics: a case–control study
Source: Front Integr Neurosci. 2024 Sep 11;18:1420339. doi: 10.3389/fnint.2024.1420339 (PMC11422087; doi:10.3389/fnint.2024.1420339)
Supplement: Supplementary file 1 [file Table_1.DOCX]

**Supplementary table 1: Neuropsychological tests and Scales results**

| **Scales and tests** | **Group** | **Mean** | **SD** | **SE** | **Cohen's d** |
| --- | --- | --- | --- | --- | --- |
| **RAVLT A1** | TC | 5.333 | 1.915 | 0.444^t^ | 0.284 |
|  | WA | 5.800 | 1.320 |  |  |
| **RAVLT A2** | TC | 8.067 | 1.534 | 0.865^MW^ | 0.082 |
|  | WA | 7.933 | 1.710 |  |  |
| **RAVLT A3** | TC | 9.867 | 1.846 | 0.702 ^t^ | 0.118 |
|  | WA | 10.067 | 1.534 |  |  |
| **RAVLT A4** | TC | 11.733 | 1.580 | 0.200^t^ | 0.48 |
|  | WA | 10.933 | 1.751 |  |  |
| **RAVLT A5** | TC | 12.467 | 1.807 | 0.069^t^ | 0.69 |
|  | WA | 11.267 | 1.668 |  |  |
| **RAVLT Total** | TC | 47.467 | 7.039 | 0.548^t^ | 0.222 |
|  | WA | 46.000 | 6.130 |  |  |
| **RAVLT B** | TC | 5.400 | 2.384 | 0.268^t^ | 0.413 |
|  | WA | 4.533 | 1.767 |  |  |
| **RAVLT A6** | TC | 10.600 | 2.098 | 0.172^t^ | 0.512 |
|  | WA | 9.467 | 2.326 |  |  |
| **RAVLT A7** | TC | 10.400 | 2.197 | 0.174^t^ | 0.509 |
|  | WA | 9.133 | 2.748 |  |  |
| **RAVLT – Recog. A** | TC | 13.533 | 1.506 | 0.580^MW^ | 0.31 |
|  | WA | 12.933 | 2.282 |  |  |
| **RAVLT – Recog. B** | TC | 7.667 | 3.658 | 0.061^t^ | 0.712 |
|  | WA | 5.200 | 3.256 |  |  |
| **TMT–A (time)** | TC | 37.333 | 9.693 | 0.693^MW^ | 0.196 |
|  | WA | 39.467 | 11.951 |  |  |
| **TMT–A (errors)** | TC | 0.133 | 0.352 | 1.000^MW^ | 0 |
|  | WA | 0.133 | 0.352 |  |  |
| **TMT-B (time)** | TC | 85.867 | 25.581 | 0.771^MW^ | 0.159 |
|  | WA | 93.600 | 63.953 |  |  |
| **TMT-B (erros)** | TC | 1.867 | 2.200 | 0.937^MW^ | 0.029 |
|  | WA | 1.933 | 2.404 |  |  |
| **DS-Forward** | TC | 5.600 | 1.404 | 0.299^MW^ | 0.359 |
|  | WA | 5.133 | 1.187 |  |  |
| **DS-Backward** | TC | 4.467 | 1.302 | 0.175^MW^ | 0.572 |
|  | WA | 3.800 | 1.014 |  |  |
| **Digit Symbol** | TC | 63.133 | 13.458 | 0.339^MW^ | 0.39 |
|  | WA | 56.267 | 20.968 |  |  |
| **Verbal Fluency (COWA)-C** | TC | 13.333 | 3.619 | 0.388^t^ | 0.32 |
|  | WA | 14.467 | 3.461 |  |  |
| **Verbal Fluency (COWA)-F** | TC | 13.533 | 4.502 | 0.634^t^ | 0.176 |
|  | WA | 14.200 | 2.908 |  |  |
| **Verbal Fluency (COWA)-L** | TC | 12.533 | 3.441 | 0.737^t^ | 0.124 |
|  | WA | 12.067 | 4.061 |  |  |
| **Verbal Fluency (COWA)-Total** | TC | 40.067 | 11.010 | 0.846^t^ | 0.071 |
|  | WA | 40.733 | 7.304 |  |  |
| **SCWT-W** | TC | 87.667 | 13.356 | 0.227^t^ | 0.451 |
|  | WA | 81.533 | 13.845 |  |  |
| **SCWT-C** | TC | 60.600 | 11.716 | 0.632^t^ | 0.177 |
|  | WA | 58.600 | 10.881 |  |  |
| **SCWT-CW** | TC | 34.667 | 11.140 | 0.563^t^ | 0.214 |
|  | WA | 32.467 | 9.372 |  |  |
| **BDI** | TC | 4.867 | 4.121 | 0.138^MW^ | 0.595 |
|  | WA | 7.600 | 5.026 |  |  |
| **BAI** | TC | 3.800 | 3.448 | 0.738^MW^ | 0.083 |
|  | WA | 4.067 | 2.963 |  |  |
| **SRQ-20** | TC | 1.467 | 1.407 | 0.264^MW^ | 0.45 |
|  | WA | 2.200 | 1.821 |  |  |
| **PSQI** | TC | 5.133 | 2.356 | 0.933^MW^ | 0.029 |
|  | WA | 5.200 | 2.274 |  |  |

^t^: Student t test. ^MW^: Mann-Whitney Test. TC: Tai Chi Group. WA: Wate Aerobics Group. BMI: Body Mass Index. RAVLT: Rey's Auditory Verbal Learning Test. BDI: Beck Depression Inventory. BAI: Beck Anxiety Inventory. TMT-A: Trail Making Test Part A. TMT-B: Trail Making Test Part B. WHO-WB: World Health Organization Well-Being. DS: Digit-Symbol. MMES: Mini-Mental State Examination. SCWT–C: Stroop Color and Word Test - Color. SCWT–W: Stroop Color and Word Test – Word. SCWT-CW: Stroop Color and Word Test - Color and Word. PSQI: Pittsburgh Sleep Quality Index: SRQ-20: Self-Report Questionnaire-20, DS: Digit Span.
