# Supplementary material for: Differences in brain connectivity between older adults practicing Tai Chi and Water Aerobics: a case–control study
Source: Front Integr Neurosci. 2024 Sep 11;18:1420339. doi: 10.3389/fnint.2024.1420339 (PMC11422087; doi:10.3389/fnint.2024.1420339)
Supplement: Supplementary file 2 [file Table_2.DOCX]

**Supplementary Material- Table 2: Descriptive behavioral data for N-back task**

|  |  | **Response Time (in ms)** | | | |  | **Accuracy** | | |  |
| --- | --- | --- | --- | --- | --- | --- | --- | --- | --- | --- |
|  |  |  |  | | **Group comparison** | |  |  | **Group comparison** | |
| **Stimulus** | **Group** | **Mean** | **SD** | **p-value** | | **Cohens' D** | **Mean** | **SD** | **p-value** | **Cohens' D** |
| **0-Back** | WA | 575.989 | 193.147 | | 0.844 | 0.070 | 0.916 | 0.116 | 0.728 | 0.147 |
|  | TAICHI | 565.709 | 74.890 | |  |  | 0.889 | 0.232 |  |  |
|  |  |  |  | |  |  |  |  |  |  |
| **2-Back** | WA | 701.323 | 151.046 | | 0.582 | 0.200 | 0.611 | 0.256 | 0.207 | 0.551 |
|  | TAICHI | 667.877 | 181.245 | |  |  | 0.749 | 0.245 |  |  |

The table shows the descriptive statistics for the N-Back task, on the accuracy and response time metrics. WA refers to Water Aerobics group. The p values denote the comparison between groups derived from the post-hoc ANOVA analysis. P-value adjusted by comparing a family of 6 corrected by the Holm-Bonferroni method. RT: Response time, in milliseconds. SD: Standard Deviation
