# Supplementary material for: Differences in brain connectivity between older adults practicing Tai Chi and Water Aerobics: a case–control study
Source: Front Integr Neurosci. 2024 Sep 11;18:1420339. doi: 10.3389/fnint.2024.1420339 (PMC11422087; doi:10.3389/fnint.2024.1420339)
Supplement: Supplementary file 3 [file Table_3.DOCX]

**Supplementary table 3: Descriptive behavioral data for Stroop task**

|  |  | **Response Time (in ms)** | | |  | **Accuracy** | | |  |
| --- | --- | --- | --- | --- | --- | --- | --- | --- | --- |
|  |  |  |  | **Group comparison** | |  |  | **Group comparison** | |
| **Stimulus** | **Group** | **Mean** | **SD** | **p-value** | **Cohen's D** | **Mean** | **SD** | **p-value** | **Cohen's D** |
| **Congruent** | WA | 756.539 | 111.141 | 1.00 | 0.068 | 0.928 | 0.124 | 1.00 | 0.426 |
|  | TAICHI | 748.342 | 127.836 |  |  | 0.967 | 0.037 |  |  |
|  |  |  |  |  |  |  |  |  |  |
| **Incongruent** | WA | 848.796 | 105.550 | 0.423 | 0.629 | 0.792 | 0.281 | 1.00 | 0.283 |
|  | TAICHI | 932.850 | 156.824 |  |  | 0.854 | 0.131 |  |  |
|  |  |  |  |  |  |  |  |  |  |
| **Neutral** | WA | 791.597 | 126.852 | 1.00 | 0.011 | 0.935 | 0.111 | 1.00 | 0.343 |
|  | TAICHI | 789.994 | 151.602 |  |  | 0.964 | 0.044 |  |  |
| The table shows the descriptive statistics for the Stroop task on the accuracy and response time metrics.  WA refers to Water Aerobics group. The p values denote the comparison between groups from the post-hoc ANOVA analysis. Adjusted p value by comparing a family of 15 corrected by the Holm-Bonferroni method. RT: Response time, in miles seconds. SD: Standard Deviation. | | | | | | | | |  |
